# Radiomics for identifying lung adenocarcinomas with predominant lepidic growth manifesting as large pure ground-glass nodules on CT images

**DOI:** 10.1371/journal.pone.0269356

**Published:** 2022-06-24

**Authors:** Ziqi Xiong, Yining Jiang, Di Tian, Jingyu Zhang, Yan Guo, Guosheng Li, Dongxue Qin, Zhiyong Li

**Affiliations:** 1 Department of Radiology, the First Affiliated Hospital of Dalian Medical University, Dalian, Liaoning, China; 2 GE Healthcare, Beijing, China; 3 Department of Pathology, the First Affiliated Hospital of Dalian Medical University, Dalian, Liaoning, China; 4 Department of Radiology, the Second Hospital of Dalian Medical University, Dalian, Liaoning, China; Universita degli Studi di Perugia, ITALY

## Abstract

**Purpose:**

To explore the value of radiomics in the identification of lung adenocarcinomas with predominant lepidic growth in pure ground-glass nodules (pGGNs) larger than 10 mm.

**Methods:**

We retrospectively analyzed CT images of 204 patients with large pGGNs (≥ 10 mm) pathologically diagnosed as minimally invasive adenocarcinomas (MIAs), lepidic predominant adenocarcinomas (LPAs), and non-lepidic predominant adenocarcinomas (NLPAs). All pGGNs in the two groups (MIA/LPA and NLPA) were randomly divided into training and test cohorts. Forty-seven patients from another center formed the external validation cohort. Baseline features, including clinical data and CT morphological and quantitative parameters, were collected to establish a baseline model. The radiomics model was built with the optimal radiomics features. The combined model was developed using the rad_score and independent baseline predictors. The performance of the models was evaluated using the area under the receiver operating characteristic curve (AUC) and compared using the DeLong test. The differential diagnosis performance of the models was compared with three radiologists (with 20+, 10+, and 3 years of experience) in the test cohort.

**Results:**

The radiomics (training AUC: 0.833; test AUC: 0.804; and external validation AUC: 0.792) and combined (AUC: 0.849, 0.820, and 0.775, respectively) models performed better for discriminating than the baseline model (AUC: 0.756, 0.762, and 0.725, respectively) developed by tumor location and mean CT value of the whole nodule. The DeLong test showed that the AUCs of the combined and radiomics models were significantly increased in the training cohort. The highest AUC value of the radiologists was 0.600.

**Conclusion:**

The application of CT radiomics improved the identification performance of lung adenocarcinomas with predominant lepidic growth appearing as pGGNs larger than 10 mm.

## Introduction

Since the International Association for the Study of Lung Cancer/American Thoracic Society/European Respiratory Society (IASLC/ATS/ERS) classification in 2011 defined the terms “lepidic predominant adenocarcinoma (LPA)” and “minimally invasive adenocarcinoma (MIA, ≤ 5 mm invasion in greatest dimension)”, many studies have started to discuss the specificity of the lepidic growth pattern [1]. As two subtypes of lung adenocarcinomas with predominant lepidic growth, MIA and LPA have a 5-year disease-free survival (DFS) rate of nearly 100%, while non-lepidic predominant adenocarcinoma (NLPA, including acinar, papillary, micropapillary, and solid) has a maximum 5-year DFS rate of 82.4% with higher risk [2].

At the molecular level, the intratumor genetic heterogeneity differed between MIA/LPA and NLPA [3]. Previous studies have shown that mutant epidermal growth factor receptor (EGFR) is associated with MIA/LPA [4, 5]. In addition, Miyazawa et al. reported that programmed death-ligand 1 (PD-L1)-positive cases were 0/12 in MIA and 1/10 in LPA, and were >1/2 in all types of NLPA [6]. For the treatment plan, MIA/LPA may be treated with limited resection (segmentectomy or wedge resection), whereas other subtypes require standard therapy for lobectomy [1, 7]. However, currently, thoracic surgeons rely heavily on the pathological assessment of intraoperative frozen sections (FS) when choosing the procedure. Some studies have pointed to the limited sensitivity of FS diagnosis for MIA/LPA [8, 9]. Hence, there is an urgent need to establish more effective, noninvasive methods to precisely identify the pathological type of invasive lesions prior to surgery to analyze their proliferation and growth status and help decide the surgical approach.

Previous studies have suggested that MIA and LPA are more likely to present as pure ground-glass nodules (pGGNs) [1, 10]. However, Son et al. reviewed CT images and pathologic specimens from 191 pGGNs, and their sample comprised 61 cases of MIA (31.94%), 49 cases of LPA (25.65%), and 43 cases of NLPA (22.51%) [11]. Additionally, in a study of stage I invasive lung adenocarcinoma by Fu et al., there were 146 cases in the pGGN group, including 81 cases of LPA (55.48%) and 64 cases of NLPA (43.84%) [12]. In other words, not only MIA and LPA but also NLPA can manifest as pGGNs [13]. Many studies have shown that invasive pGGNs are larger than or equal to 10 mm in size [14, 15]. In a previous study, the likelihood of invasive lesions was 88.73% when the size was greater than 10.5 mm [16]. In addition, a previous study showed that invasive lung adenocarcinomas larger than 10 mm are more likely to be misdiagnosed by FS due to sampling errors [8]. Based on these studies, we determined a threshold of 10 mm for large pGGNs.

Medical images implicitly contain many high-throughput data that cannot be identified by the naked eye [17]. Radiomics can capture tumor heterogeneity and quantify the characteristics of tumor appearance, structure, and arrangement into texture features to better characterize the tumor environment from medical images, and it has been widely used in studies related to lung malignancies [18, 19]. Our objectives were to use radiomics to fully exploit the information in CT images to distinguish between MIA/LPA and NLPA appearing as pGGNs larger than 10 mm and to compare the predictive performance of the radiomics model with a baseline model with clinical data, CT morphological and quantitative parameters, and the integrated baseline-radiomics combined model.

## Materials and methods

### Patients

This retrospective study was approved by the Ethics Committee of the First Affiliated Hospital of Dalian Medical University and the Ethics Committee of the Second Hospital of Dalian Medical University, and the requirement for informed consent was waived.

We reviewed patients with lung adenocarcinomas that had postoperative pathological results and presented as pGGNs on imaging at center 1 between November 2012 and December 2018. The inclusion criterion was invasive lesions from adenocarcinoma with pGGNs ≥ 10 mm. The exclusion criteria were (i) no CT images with a slice thickness of 1.5 mm or less within one month before surgery; (ii) biopsy, radiotherapy, chemotherapy, or surgical resection of nodules before CT scan; and (iii) significant artifacts around nodules on CT images. In our study, pGGNs were defined as lung nodules without any solid component in the mediastinal window setting (level, 40 HU; width, 400 HU), while one of their first-order histogram features, 90th percentile, needed to be less than or equal to –300 HU [15, 20]. In addition, we collected pGGNs with surgically confirmed lung adenocarcinoma from April 2021 to January 2022 from center 2, and eligible lesions were identified using the same inclusion and exclusion criteria. Since the number of NLPAs was smaller than that of MIAs and LPAs, we used a simple random undersampling method in datasets of center 1 to obtain the same number of MIAs and LPAs and adjusted the ratio of the sample sizes in the MIA/LPA and NLPA groups to 1. This helped to eliminate the effect of sample imbalance and ensure that our modeling and validation were based on actual data.

### Pathological evaluation

A senior pathologist blinded to the previous diagnoses reviewed the hematoxylin and eosin-stained slides of all surgically resected specimens to reconfirm the pathologic diagnosis. The outcome was based on the adenocarcinoma classification proposed by IASLC/ATS/ERS [1]. For invasive adenocarcinomas (IAs), we used comprehensive histological subtyping to assess histological type in 5% increments. The histological subtypes were divided into two groups based on the lepidic status (MIA/LPA and NLPA).

In addition, FS pathology results were collected for further analysis. The results were diagnosed by pathologists immediately after tumors were removed and reported according to the adenocarcinoma classification proposed by IASLC/ATS/ERS [1].

### Baseline features

The clinical data included sex, age, and smoking status. Two thoracic radiologists (a junior radiologist and a senior radiologist with 20 years of experience) who were blinded to each patient’s clinical and pathological information assessed the CT morphological parameters, including tumor location, shape (irregular or round and oval), tumor-lung interface (clear or unclear), lobulation (an appearance resembling lobules), vacuole (air attenuation vesicle-like lucency), air bronchogram (air-filled bronchi of low attenuation), and pleural indentation (pleural retraction, or pleural thickening at the pleural end). The CT quantitative parameters, including volume (cm^3^), maximum diameter (on the largest cross-section, cm), mean CT value of the whole nodule (mCTv, HU), mean CT value of the largest cross-section (mCTv-Lcs, HU), and mass (mg), were also recorded. The formula to calculate mass was [21]:

$$Mass\;=volume\times \left(mCTv\;+1000\right).$$

### Image acquisition

Images were obtained using several tomographs, including Optima CT660, Discovery CT750 HD, Revolution CT and LightSpeed16 (General Electric), SOMATOM Perspective and Emotion 16 (Siemens), and Brilliance 16P (Philips) with the following parameters: matrix, 512×512; in-plane pixel size, 0.6–0.9 mm; rotation time, 0.5–0.6 s; tube voltage, 120 kVp; tube current, 170–200 mA. CT imaging data were reconstructed by using the lung reconstruction algorithm with a thickness of 1–1.25 mm and slice interval of 1.00–1.50 mm. All CT examinations were performed without intravenous contrast material injection.

### Image segmentation and radiomics feature extraction

Segmentation of pGGNs was performed on CT images by a radiologist who was unaware of the pathological findings of the nodules. The regions of interest (ROIs) were plotted layer by layer on CT images with the lung window setting (level, –600 HU; width, 1500 HU), excluding the bronchi, vacuoles, and blood vessels. Then, 106 well-defined radiomics features were extracted. The open software 3Dslicer (version 4.8.1, https://www.slicer.org/) was used for image segmentation and radiomics feature extraction (S1 File).

### Feature selection and modeling

Feature selection and modeling were performed in the training cohort. Univariate and multivariate logistic regression analyses were used to select the optimal features in the clinical data, CT morphological parameters, and CT quantitative parameters, which ensured that the features in the baseline model were all independent and valid predictors.

For the radiomics features, we used inter- and intraclass correlation coefficients (ICCs), max-relevance and min-redundancy (mRMR), and least absolute shrinkage and selection operator (LASSO) methods to select the most effective radiomics features.

First, we randomly selected 40 pGGNs as small samples. Two radiologists independently segmented the pGGNs in these samples and extracted radiomics features. One of the radiologists performed the second segmentation one month later. The ICCs were calculated to evaluate the consistency and reliability of radiomics features. In our study, we retained only features with an ICC of ≥ 0.75 [22, 23]. Furthermore, after the data were standardized using StandardScaler, mRMR was used to select features according to the maximum dependency criterion and punish the correlation of features by its redundancy in the presence of other selected features. Then, LASSO can shrink the regression coefficients within a certain region and construct a first-order penalty function to obtain a refined model. With tenfold cross-validation, the best hyperparameter λ was obtained during the regularized L1 logistic regression procedure to choose the best model.

Five other machine learning methods, including support vector machine, naive Bayesian classifier, K-nearest neighbor, decision tree, and random forest, were also used to build the model with the selected radiomics features and compare the performance to select the best model.

Finally, we combined the features of the baseline model and the rad_score to construct the combined model and plotted the nomogram.

### Performance comparison with radiologists

Three thoracic radiologists (Radiologist A, B, and C) with 20+, 10+, and 3 years of experience in thoracic imaging, respectively, were asked to make a differential diagnosis (MIA, LPA, or NLPA) of the cases in the test cohort.

### Statistical analysis

We used receiver operator characteristic (ROC) curve analysis to observe the performance of the models and calculated the corresponding area under the curve (AUC), 95% confidence interval (CI), sensitivity, specificity, and accuracy for the two cohorts. The DeLong test was used to compare the AUCs to visualize whether there was a significant improvement in model performance and to verify the stability of the models between the training and test cohorts. In addition, we observed the goodness of fit of the models by performing the Hosmer–Lemeshow test and plotting calibration curves. Decision curve analysis was applied to evaluate the clinical utility of the model.

Variable differences between the two groups were assessed using a traditional monofactor analysis. Differences in sex, smoking status, and CT morphological features were analyzed using a chi-square test or Fisher’s exact test. The Mann–Whitney test or Student’s t test was used for continuous variables. Statistical significance was set at *p* < 0.05. SPSS (version 26.0, IBM), R (version 3.5.1), and Python (version 3.5.6) were used for statistical analyses.

## Results

### Demographic characteristics

**Fig 1:**
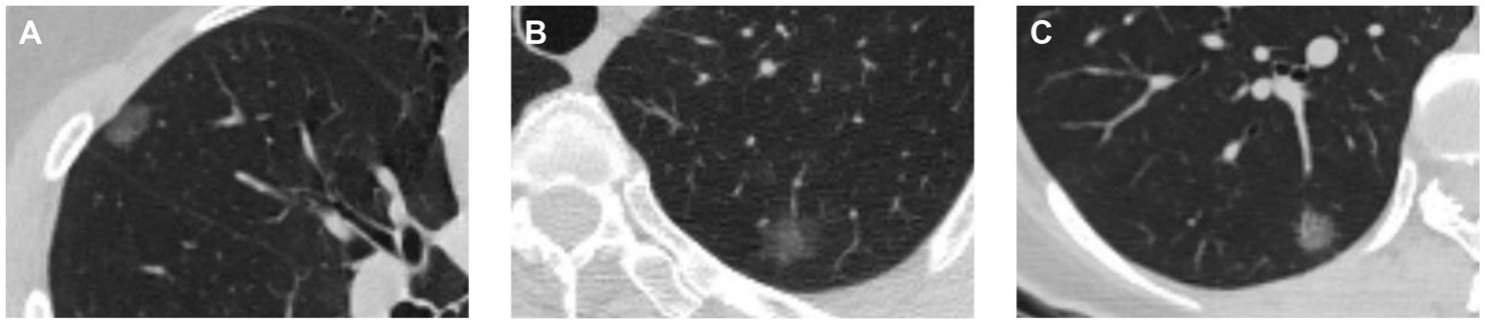
CT images and features of three pure ground-glass nodules with different pathological results. (A) A 39-year-old woman with a pure ground-glass nodule (pGGN) in the right middle lobe was diagnosed pathologically with minimally invasive adenocarcinoma (MIA). The CT image shows that the size of the pGGN is 1.34 cm. The mean CT value of the whole nodule (mCTv) was –694.21 HU, as measured using a region of interest (ROI) after 3D volume segmentation, and the rad_score was –1.340. (B) A 63-year-old woman with a pGGN in the left upper lobe was diagnosed pathologically with lepidic predominant adenocarcinomas (LPA). The CT image shows that the size of the pGGN is 1.55 cm. The mCTv was –708.496 HU, and the rad_score was –1.139. (C) A 42-year-old woman with a pGGN in the right lower lobe was diagnosed pathologically with non-lepidic predominant adenocarcinoma (NLPA). The CT image shows that the size of the pGGN is 1.22 cm. The mCTv was –566.138 HU, and the rad_score was 0.262.

Two hundred and four patients (68 men and 136 women; median age, 61 years [interquartile range, 55–66 years]) were included in center 1. A total of 204 pGGNs from these patients included 51 MIAs, 51 LPAs, and 102 NLPAs. Patients in the two groups (MIA/LPA and NLPA) were randomized in a 7:3 ratio into two separate cohorts for training and testing. Examples of these cases are presented in Fig 1.

Forty-nine pGGNs of 47 patients (16 men and 31 women; mean age, 59.51 ± 10.68 years) were collected as the external validation cohort for performance testing. There were two patients with two pGGNs (all pathologically diagnosed as MIAs) and forty-five patients with a single pGGN, including 15 MIAs, 14 LPAs, and 16 NLPAs.

### Model construction

A comparative analysis of variables between MIA/LPA and NLPA in the two cohorts from center 1 is shown in Table 1. There were significant differences in mCTv and mCTv-Lcs in both the training and test cohorts. However, after the univariate and multivariate logistic regression analyses in the training cohort, only tumor location (*p* = 0.014) and mCTv (*p* < 0.001) remained significant (Table 2).

**Table 1:** Comparison of variables between MIA/LPA and NLPA in the training and test cohorts.

Variables	Training cohort			Test cohort		
	MIA/LPA	NLPA	*p* value	MIA/LPA	NLPA	*p* value
**Age (year)**	58.96 ± 11.40	61.00 (57.00, 65.75)	0.511	62.00 ± 10.48	60.93 ± 7.34	0.650
**Sex**			0.289			0.584
Female	45 (62.50%)	51 (70.83%)		19 (63.33%)	21 (70.00%)	
Male	27 (37.50%)	21 (29.17%)		11 (36.67%)	9 (30.00%)	
**Smoking status**			0.413			0.353
Never smoked	63 (87.50%)	66 (91.67%)		29 (96.67%)	26 (86.67%)	
Former or current smoker	9 (12.50%)	6 (8.33%)		1 (3.33%)	4 (13.33%)	
**Tumor location**			0.099			0.318
Left lower lobe	15 (20.83%)	7 (9.72%)		3 (10.00%)	4 (13.33%)	
Left upper lobe	22 (30.56%)	16 (22.22%)		10 (33.33%)	7 (23.33%)	
Right lower lobe	8 (11.11%)	13 (18.06%)		6 (20.00%)	4 (13.33%)	
Right middle lobe	4 (5.56%)	2 (2.78%)		0 (0.00%)	4 (13.33%)	
Right upper lobe	23 (31.94%)	34 (47.22%)		11 (36.67%)	11 (36.67%)	
**Shape**			0.404			0.067
Irregular	36 (50.00%)	31 (43.06%)		16 (53.33%)	9 (30.00%)	
Round and oval	36 (50.00%)	41 (56.94%)		14 (46.67%)	21 (70.00%)	
**Tumor-lung interface (clear)**			0.695			0.117
Clear	54 (75.00%)	56 (77.78%)		21 (70.00%)	26 (86.67%)	
Unclear	18 (25.00%)	16 (22.22%)		9 (30.00%)	4 (13.33%)	
**Lobulation**			0.849			1.000
Presence	54 (73.61%)	53 (73.61%)		22 (73.33%)	22 (73.33%)	
Absent	18 (26.39%)	19 (26.39%)		8 (26.67%)	8 (26.67%)	
**Vacuole**			0.853			0.781
Presence	20 (27.78%)	21 (29.17%)		9 (30.00%)	10 (33.33%)	
Absent	52 (72.22%)	51 (70.83%)		21 (70.00%)	20 (66.67%)	
**Air bronchogram**			0.846			0.766
Presence	17 (23.61%)	18 (25.00%)		7 (23.33%)	8 (26.67%)	
Absent	55 (76.39%)	54 (75.00%)		23 (76.67%)	22 (73.33%)	
**Pleural indentation**			0.613			0.584
Presence	29 (40.28%)	32 (44.44%)		9 (30.00%)	11 (36.67%)	
Absent	43 (59.72%)	40 (55.56%)		21 (70.00%)	19 (63.33%)	
**Volume (cm** ^**3**^ **)**	1.02 (0.53, 1.61)	1.07 (0.66, 2.06)	0.453	1.22 (0.60, 2.84)	1.01 (0.69, 1.62)	0.433
**Maximum diameter (cm)**	1.56 (1.23, 1.80)	1.55 (1.35, 2.07)	0.207	1.82 ± 0.57	1.52 (1.36, 2.12)	0.579
**mCTv (HU)**	–674.82 ± 59.24	–610.52 (-682.99, -577.62)	<0.001	–669.99 ± 60.41	–611.83 ± 58.96	<0.001
**mCTv-Lcs (HU)**	–664.99 ± 70.61	–605.69 ± 72.26	<0.001	–658.41 ± 72.77	–602.03 ± 76.72	0.005
**Mass (mg)**	314.33 (177.48, 499.58)	384.47 (222.31, 702.13)	0.060	373.06 (218.80, 958.132)	399.86 (263.62, 611.24)	0.929

The values are presented as no. (%), mean ± standard deviation, or median (interquartile range). MIA, minimally invasive adenocarcinoma; LPA, lepidic predominant adenocarcinoma; NLPA, non-lepidic predominant adenocarcinoma; mCTv, mean CT value of the whole nodule; mCTv-Lcs, mean CT value of the largest cross-section; HU, Hounsfield units.

**Table 2:** Univariate and multivariate logistic regression analysis for the baseline features.

Features	Univariate Logistic Regression			Multivariate Logistic Regression		
	OR	95% CI	*p* value	OR	95% CI	*p* value
**Tumor location**	1.276	1.030, 1.582	0.026	1.351	1.064, 1.716	0.014
**mCTv**	1.014	1.007, 1.020	<0.001	1.014	1.008, 1.021	<0.001
**mCTv-Lcs**	1.009	1.006, 1.017	<0.001			

mCTv, mean CT value of the whole nodule; mCTv-Lcs, mean CT value of the largest cross-section; OR, odds ratio; CI, confidence interval.

According to the three feature selection methods, we removed unstable radiomics features and chose the best nine features to construct the final model (further details about the nine radiomics features are given in S2 File). The mean (standard deviation) inter- and intraclass ICC values of the final nine features were 0.870 (0.061) and 0.919 (0.066), respectively. After comparing the performance of the models with six machine learning methods (S1 Table), the best logistic regression was used to build the radiomics model and generate the rad_score, which was created by summing the selected features weighted by their coefficients. The other five methods were discarded because of apparent differences in performance or the presence of overfitting problems in the two cohorts.

The formula to calculate rad_score was:

$$\begin{array}{ll} Rad\_score & =-0.047+0.225*10Percentile+0.457*glcmMCC-0.015\\ & *gldmSmallDependenceLowGrayLevelEmphasis+0.324\\ & *Maximum2DDiameterSlice+0.321*Maximum-0.509\\ & *Skewness-0.29*Sphericity-0.96\\ & *glszmLargeAreaHighGrayLevelEmphasis+0.189\\ & *glszmZoneEntropy \end{array}$$

The boxplots of the radiomics model are shown in S1 Fig.

**Fig 2:**
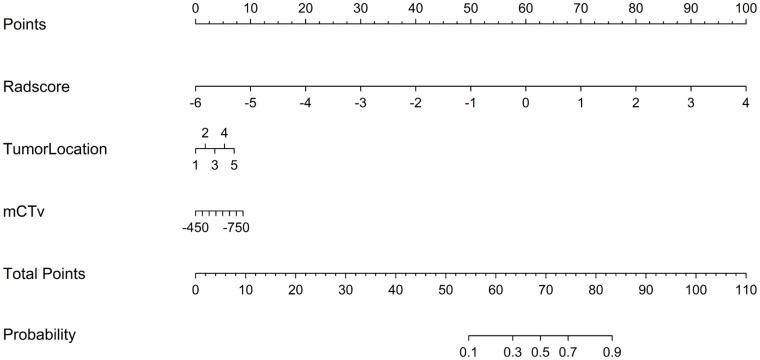
Nomogram of the combined model for the training cohort. The personalized predictive nomogram was constructed with tumor location, mean CT value of the whole nodule (mCTv), and rad_score. The “1”—“5” in the diagram represents the tumor location in the left lower lobe, left upper lobe, right lower lobe, right middle lobe, and right upper lobe.

**Fig 3:**
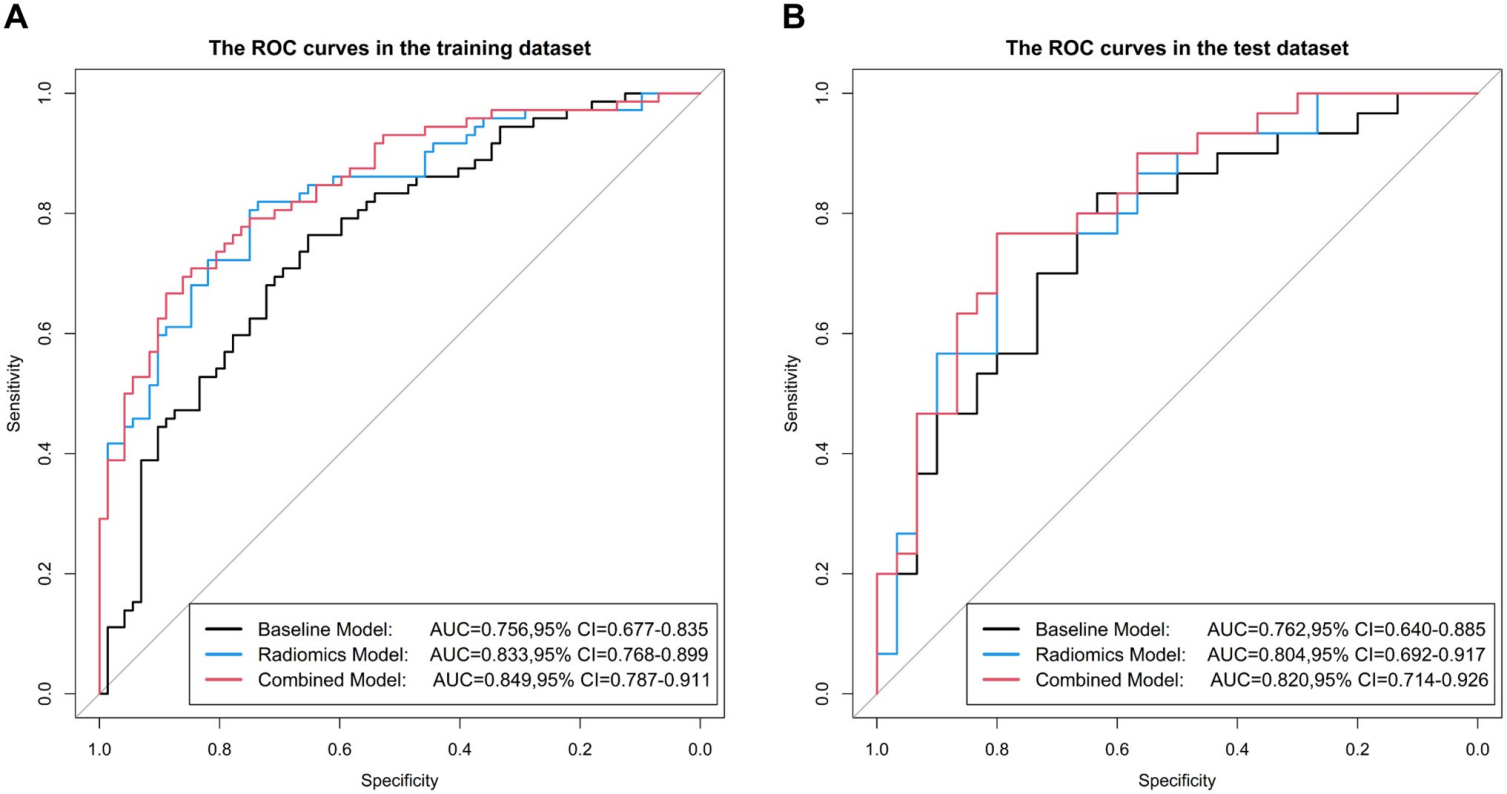
Receiver Operating Characteristic (ROC) curves of the three models for the training (A) and test (B) cohorts. AUC, area under the receiver operating characteristic curve; CI, confidence interval.

The model that combined the tumor location, mCTv, and rad_score was developed as a nomogram (Fig 2). The ROC curves of the three models are shown in Fig 3, and the corresponding AUC and other performance parameters are presented in Table 3. The radiomics (training AUC, 0.833; test AUC, 0.804) and combined (AUC, 0.849 and 0.820, respectively) models performed better for discriminating than the baseline model (AUC, 0.756 and 0.762, respectively).

**Table 3:** Performance of the three prediction models.

	AUC	95% CI	SEN	SPE	ACC
**Training cohort**					
Baseline model	0.756	0.677, 0.835	0.687	0.734	0.708
Radiomics model	0.833	0.768, 0.899	0.736	0.819	0.778
Combined model	0.849	0.787, 0.911	0.823	0.744	0.778
**Test cohort**					
Baseline model	0.762	0.640, 0.885	0.694	0.792	0.733
Radiomics model	0.804	0.692, 0.917	0.800	0.766	0.783
Combined model	0.820	0.714, 0.926	0.793	0.774	0.783

AUC, area under the receiver operating characteristic curve; CI, confidence interval; SEN, sensitivity; SPE, specificity; ACC, accuracy.

### Performance evaluation

**Fig 4:**
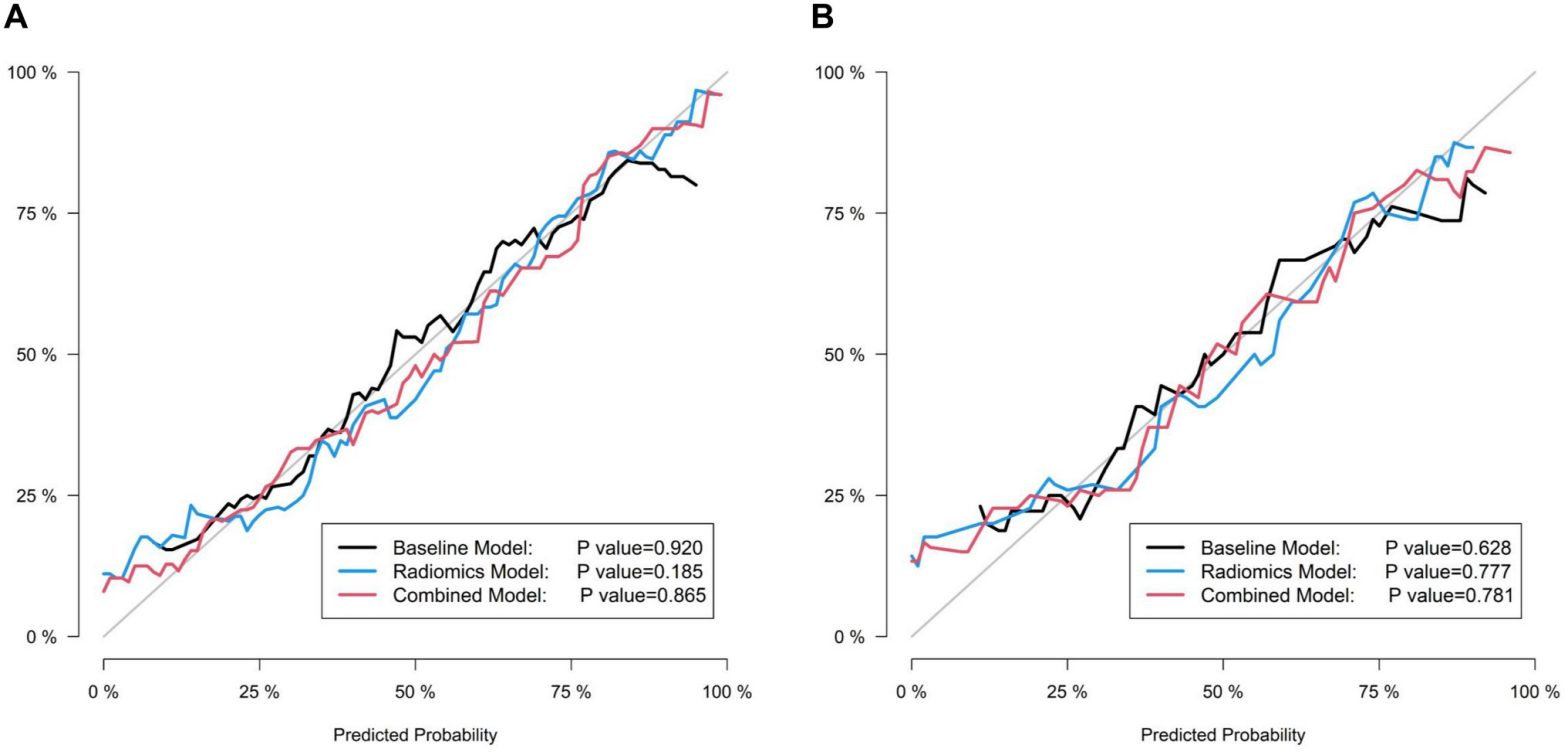
Calibration curves of the three models for the training (A) and test (B) cohorts.

**Fig 5:**
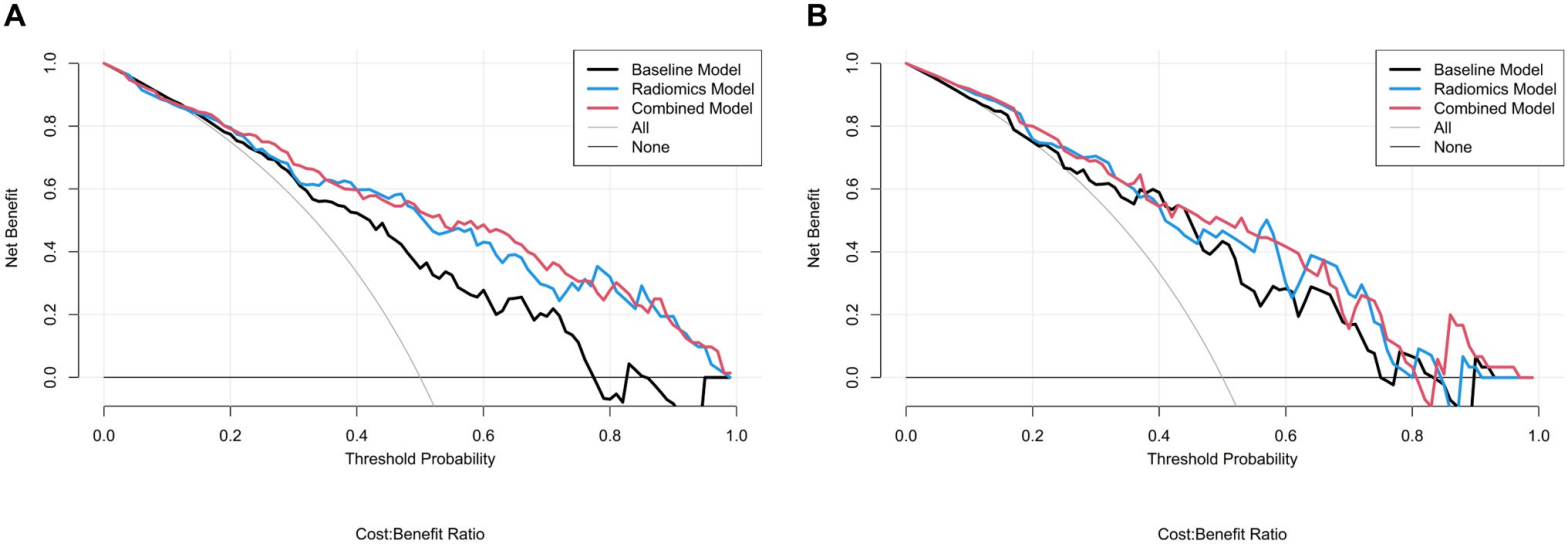
Decision curves of the three models for the training (A) and test (B) cohorts.

According to the ROC curves, the area under the curve for the radiomics and combined models was significantly larger than that of the baseline model, demonstrating the improved performance due to radiomics. The DeLong test also showed that the AUC values of the radiomics model and the combined model were significantly better than that of the baseline model in the training cohort (*p* = 0.015 and 0.002, respectively). In addition, the models had no significant difference in AUC values between the training and test cohorts, demonstrating that none of the three models had any overfitting problems. The calibration curves (Fig 4) and the results of the Hosmer–Lemeshow test showed that all three models had good agreement with the actual observations in the two cohorts (*p* > 0.05, all). The decision curves are plotted in Fig 5.

Additionally, the radiomics model also showed superior diagnostic performance (AUC, 0.792) in the external validation cohort, especially for the sensitive identification of NLPA (Table 4). The baseline features of the external validation cohort are shown in S2 Table.

**Table 4:** Performance of the three prediction models in the external validation cohort.

	AUC	95% CI	SEN	SPE	ACC
**External validation cohort**					
Baseline model	0.725	0.577, 0.874	0.500	0.667	0.612
Radiomics model	0.792	0.668, 0.916	0.875	0.576	0.673
Combined model	0.775	0.644, 0.905	0.938	0.515	0.653

AUC, area under the receiver operating characteristic curve; CI, confidence interval; SEN, sensitivity; SPE, specificity; ACC, accuracy.

### Diagnostic performance of the radiologists

The differential diagnosis performance of the radiologists can be found in Table 5. The highest AUC value of the radiologists was 0.600.

**Table 5:** The differential diagnosis performance of the radiologists.

	AUC	95% CI	SEN	SPE	ACC
Radiologist A	0.600	0.456, 0.744	0.600	0.600	0.600
Radiologist B	0.500	0.353, 0.647	0.167	0.833	0.500
Radiologist C	0.550	0.403, 0.697	0.267	0.833	0.550

Radiologists A, B and C are radiologists with 20+, 10+, and 3 years of experience in thoracic imaging. AUC, area under the receiver operating characteristic curve; CI, confidence interval; SEN, sensitivity; SPE, specificity; ACC, accuracy.

### Frozen section diagnosis

Since the pathologists at our institution did not identify LPA and NLPA by FS, the FS results of the 204 cases in this study included adenocarcinoma in situ (AIS), MIA, and IA. A comparison of FS and final pathology diagnosis is shown in S3 Table.

## Discussion

In our study, radiomics features extracted from CT images were used to better discriminate NLPA from MIA/LPA appearing as large pGGNs compared to clinical data and traditional CT parameters. In the training cohort, the differential diagnostic performance of the combined model (AUC value improved by 0.093) and the radiomics model (AUC value improved by 0.077) was significantly improved compared to the baseline model, and this prediction ability was consistently achieved in the test and external validation cohorts.

As a specific subgroup of lung nodules, pGGN has been the focus of research regarding its invasiveness, prognosis, and follow-up. The Fleischner Society Guidelines state that a ground-glass nodule manifests as hazy increased attenuation in the lung that does not obliterate the bronchial and vascular margins [24]. However, the definition and differentiation of pGGNs remain unclear. Therefore, in our study, we utilized both qualitative and quantitative criteria to ensure that the pGGNs used for modeling were reliable.

Some studies have differed on the issue of the value of CT density in identifying LPA and NLPA [25–27]. The study of Fu et al. showed that tumor size (OR: 5.316, *p* < 0.001) was the only independent predictor of NLPA instead of CT density [27]. In our study, multivariate logistic regression analysis identified mCTv as an independent predictor of NLPA. This may be because our tumor density measurements were based on 3D volume segmentation, whereas the measurements in their study were not. Our results also showed that the maximum diameter measured using the same method was not significantly different between the two groups, probably because the artificial measurement of this parameter for pGGNs may be influenced by the experience of observers. In addition, since the object of our study was pGGNs larger than 10 mm, the effect of size on the differential diagnosis between the two groups was eliminated to a certain extent.

A previous study used quantitative measurement parameters and first-order histogram features to build logistic regression models to distinguish between MIA/LPA and NLPA [28]. The AUC of their model based on size and mean nodule attenuation in the third quartile of the first-order histogram yielded an AUC of 0.877. Eriguchi et al. found that the 75th percentile (*p* < 0.001) and the maximum (*p* = 0.009) in first-order histogram features were associated with NLPA [29]. Our experiments analyzed a wider range of radiomics features, particularly higher-order texture features, which could quantify heterogeneity. The diversity of tumor histopathology and the presence of multiple subclones within the tumor make it heterogeneous, and high intratumoral heterogeneity may be associated with a higher tumor grade [30]. In addition, Katsumata et al. showed that LPA was characterized by a less invasive component of pro-oncogenic mesenchymal cells [31]. This indicates that the invasive component of LPA is associated with a less malignant tumor microenvironment. In our study, we proved that our radiomics features could reflect the slight differences between MIA/LPA and NLPA. Park et al. used radiomics to discriminate between LPA and NLPA [32]. In their study, the radiomics model using two first-order histogram features and three texture features had an AUC value of 0.917 (95% CI, 0.894–0.939). However, their study was not based on pGGNs.

The current guidelines suggest a conservative follow-up strategy to treat pGGNs due to their usually indolent behavior [33]. However, in a previous study based on 124 patients, 51.6% of pGGNs showed growth during the two-year follow-up period, and over 40% of the tumors with growth were confirmed by postoperative pathology to be IAs, while most unchanged tumors were MIAs [34]. Additionally, a previous study found the presence of pGGNs growing faster than subsolid nodules of large size (≥ 8 mm), which could rapidly develop into the solid component [35]. The literature above suggests that pGGNs may also become clinically active adenocarcinomas and that the particulars of the invasive component are influential factors. In our study, in addition to the change in AUC, the sensitivity was clearly improved with the application of radiomics in all cohorts. Compared to the baseline model, the radiomics model and the combined model could better predict NLPA, which requires closer monitoring in pGGNs larger than 10 mm.

As seen by the radiologists’ diagnosis, even if a pGGN is large in size, the radiologists would relax their vigilance and tend to consider the nodule as low risk due to the absence of a solid component that directly represents the invasive foci. In addition, the poor diagnostic performance of radiologists further reflected the limited value of conventional CT features in discriminating between MIA/LPA and NLPA. In contrast, the radiomics model was able to more sensitively identify NLPAs that present as pGGNs, prompting clinicians to adjust the follow-up frequency and management strategies for these lesions.

Currently, some pathologists have suggested that it is necessary to identify the predominant subtypes of adenocarcinomas by FS, but this is difficult to achieve and is limited by sampling error. The study of Trejo Bittar HE et al. showed that the sensitivity of identifying LPA on FS was only 33.3% [9]. Our data also reflect the truth that it is difficult to recommend a definitive assessment of adenocarcinomas by FS alone. FS in combination with or comparison with radiology seems to be a more rational approach. Previous studies have confirmed that radiomics signatures in combination with FS can help classify peripheral lung adenocarcinoma [36–38]. In the study of Wang et al., multivariate analyses showed that the different diagnosis between FS and the radiomic model they developed was the independent predictive factor for the misdiagnosis of FS (OR: 7.46; *p* < 0.001) [36]. In our study, using the radiomics model, we achieved an AUC of 0.804 to distinguish between MIA/LPA and NLPA. Thus, our models may help pathologists and clinicians to accurately determine pathology subtypes and facilitate the selection of surgical approaches.

Our study had several limitations. First, the small sample size of the test cohort and external validation cohort may have interfered with the results of our model. We will further confirm the generalizability of our model with more standardized trials with large sample sizes in the future. In the meantime, we should require the same CT scanning machine or standardization of scanning protocols or use image resampling and batch effect correction to minimize acquisition-related radiomics variability and thus improve the robustness of the models. Second, we should have included more features for modeling, such as peritumor radiomics features. Our study only targeted lung adenocarcinomas in pGGNs, but in the clinic, there are still many benign lesions or precursor glandular lesions that present as pGGNs, and they require advanced methods to enable their identification on early images to avoid overdiagnosis. In subsequent studies, we will establish a multiclass classification model and provide a more comprehensive diagnostic approach to the clinic.

In conclusion, rather than relying on the clinical, CT morphological, and CT quantitative features used in the past, the application of radiomics, a noninvasive and efficient approach, can describe pGGNs larger than 10 mm from CT images more accurately and help clinicians achieve risk stratification, thus providing a more targeted treatment strategy for each patient.

## Supporting information

S1 File Radiomics feature extraction. (DOCX)

S2 File Detailed information about the final 9 radiomics features. (DOCX)

S1 Fig Boxplots of the radiomics model in the training (A) and test (B) cohorts. (TIF)

S1 Table Prediction performance of radiomics models based on six machine learning methods. (DOCX)

S2 Table Comparison of CT morphological and quantitative parameters between MIA/LPA and NLPA in the external validation cohort. (DOCX)

S3 Table Comparison of frozen sections and final pathology diagnosis. (DOCX)
