# Supplementary material for: Radiomics for identifying lung adenocarcinomas with predominant lepidic growth manifesting as large pure ground-glass nodules on CT images
Source: PLoS One. 2022 Jun 24;17(6):e0269356. doi: 10.1371/journal.pone.0269356 (PMC9231804; doi:10.1371/journal.pone.0269356)
Supplement: S2 File — (DOCX) [file pone.0269356.s002.docx]

**Detailed information about the final nine radiomics features**

**1) Shape-based features**

Shape-based features describe the size and shape of a given ROI, without taking into account the attenuation values of its voxels. These were defined as follows:

**Let:**

**V** the volume of the ROI in mm^3^

**A** the surface area of the ROI in mm^2^

**Table S1. Detailed information about the shape-based features in the final 9 radiomics features.**

| **Radiomic feature** | **Interpretation** | |
| --- | --- | --- |
| **Maximum 2D diameter (Slice)** | | Maximum 2D diameter (Slice) is defined as the largest pairwise Euclidean distance between ROI surface voxels in the row-column (generally the axial) plane. |
| $\text{Sphericity=}\frac{\sqrt[\text{3}]{\text{36π}\text{V}^{\text{2}}}}{\text{A}}$ | | Sphericity is a measure of the roundness of the shape of the tumor region relative to a sphere. It is a dimensionless measure, independent of scale and orientation. The value range is 0<sphericity≤1, where a value of 1 indicates a perfect sphere (a sphere has the smallest possible surface area for a given volume, compared to other solids). |
| Adapted from http://pyradiomics.readthedocs.io/en/latest/features.html. | | |

**2) First-order histogram features**

First-order statistics describe the distribution of voxel intensities within the image region defined by the mask through commonly used and basic metrics., as follows:

**Let:**

***X*** be a set of ***N_p_*** voxels included in the region of interest (ROI)

***P(i)*** be the first order histogram with ***N_g_***discrete intensity levels, where ***N_g_*** is the number of non-zero bins, equally spaced from 0 with a width.

***p(i)*** be the normalized first order histogram and equal to $\frac{\text{P}\boldsymbol{(i)}}{\boldsymbol{N}_{\boldsymbol{p}}}$

**Table S2.** **Detailed information about the first-order histogram features in the final 9 radiomics features.**

| **Radiomic feature** | **Interpretation** | |
| --- | --- | --- |
| **10 percentile** | | The 10th percentile of X |
| $\text{Maximum}=max(\text{X})$ | | The maximum gray level intensity within the ROI. |
| $\text{Skewness=}\frac{\text{μ}_{\text{3}}}{\text{σ}^{\text{3}}}\text{=}\frac{\frac{\text{1}}{\text{N}_{\text{p}}}\sum_{\text{i=1}}^{\text{N}_{\text{p}}} \text{(X(i)-}\overline{\text{X}}\text{)}^{\text{3}}}{{\text{(}\sqrt{\frac{\text{1}}{\text{N}_{\text{p}}}\sum_{\text{i=1}}^{\text{N}_{\text{p}}} \text{(X(i)-}\overline{\text{X}}\text{)}^{\text{2}}}\text{)}}^{\text{3}}}$ | | Skewness measures the asymmetry of the distribution of values about the Mean value. Depending on where the tail is elongated and the mass of the distribution is concentrated, this value can be positive or negative. (Where μ3 is the 3rd central moment). |
| Adapted from http://pyradiomics.readthedocs.io/en/latest/features.html. | | |

**3) Gray Level Co-occurrence Matrix (GLCM) Features**

A GLCM of size ***N_g_***×***N_g_*** describes the second-order joint probability function of an image region constrained by the mask and is defined as P(***i,j***|***δ,θ***). The (***i,j***)^th^ element of this matrix represents the number of times the combination of levels ***i*** and ***j*** occur in two pixels in the image, that are separated by a distance of ***δ*** pixels along angle ***θ***. The distance ***δ*** from the center voxel is defined as the distance according to the infinity norm. For ***δ=1***, this results in 2 neighbors for each of 13 angles in 3D (26-connectivity) and for ***δ=2*** a 98-connectivity (49 unique angles).

**Let:**

**ϵ** be an arbitrarily small positive number **(≈2.2×10−16**)

**P(*i,j*)** be the co-occurence matrix for an arbitrary ***δ*** and ***θ***

**p(*i,j*)** be the normalized co-occurence matrix and equal to $\frac{\text{P}(i,j)}{\sum\text{P}(i,j)}$

**Ng** be the number of discrete intensity levels in the image

$\boldsymbol{p}_{\boldsymbol{x}}\boldsymbol{(i)=}\sum_{\boldsymbol{j=1}}^{\boldsymbol{N}_{\boldsymbol{g}}} \boldsymbol{P(i,j)}$ be the marginal row probabilities

$\boldsymbol{p}_{\boldsymbol{y}}\boldsymbol{(j)=}\sum_{\boldsymbol{i=1}}^{\boldsymbol{N}_{\boldsymbol{g}}} \boldsymbol{P(i,j)}$ be the marginal column probabilities

**Table S3. Detailed information about the GLCM features in the final 9 radiomics features.**

| **Radiomic feature** | **Interpretation** | |
| --- | --- | --- |
| $\boldsymbol{Maximal Correlation Coefficient}\left( \boldsymbol{MCC} \right)$  $\boldsymbol{MCC=}\sqrt{\boldsymbol{second largest eigenvalue ofQ}}$  $\boldsymbol{Q}\left( \boldsymbol{I,J} \right)\boldsymbol{=}\sum_{\boldsymbol{k=0}}^{\boldsymbol{N}_{\boldsymbol{g}}} \frac{\boldsymbol{p(i, k)p(j,k)}}{\boldsymbol{p}_{\boldsymbol{x}}\boldsymbol{(i)}\boldsymbol{p}_{\boldsymbol{y}}\boldsymbol{(k)}}$ | | The MCC is a measure of complexity of the texture and 0≤MCC≤1.  In case of a flat region, each GLCM matrix has shape (1, 1), resulting in just 1 eigenvalue. In this case, an arbitrary value of 1 is returned. |
| Adapted from http://pyradiomics.readthedocs.io/en/latest/features.html. | | |

**4) Gray Level Dependence Matrix (GLDM) Features**

A Gray Level Dependence Matrix (GLDM) quantifies gray level dependencies in an image. A gray level dependency is defined as the number of connected voxels within distance ***δ*** that are dependent on the center voxel. A neighbouring voxel with gray level ***j*** is considered dependent on center voxel with gray level ***i*** if ***|i−j|≤α***. In a gray level dependence matrix ***P(i,j)*** the ***(i,j)^th^*** element describes the number of times a voxel with gray level ***i*** with ***j*** dependent voxels in its neighbourhood appears in image.

**Let:**

***N_g_*** be the number of discreet intensity values in the image

***N_d_*** be the number of discreet dependency sizes in the image

***N_z_*** be the number of dependency zones in the image, which is equal to $\sum_{i=1}^{N_{g}} \sum_{j=1}^{N_{d}} \text{P}(i,j)$

***P(i,j)*** be the dependence matrix

***p(i,j)*** be the normalized dependence matrix, defined as $p(i,j)=\frac{\text{P}(i,j)}{N_{z}}$

**Table S4. Detailed information about the GLDM features in the final 9 radiomics features.**

| **Radiomic feature** | **Interpretation** | |
| --- | --- | --- |
| $\boldsymbol{Small Dependence Low Gray Level Emphasis}=\frac{\sum_{i=1}^{N_{g}} \sum_{j=1}^{N_{d}} \frac{\text{P}(i,j)}{i^{2}j^{2}}}{N_{z}}$ | | The Small Dependence Low Gray Level Emphasis measures the joint distribution of small dependence with lower gray-level values. |
| Adapted from http://pyradiomics.readthedocs.io/en/latest/features.html. | | |

5) Gray Level Size Zone Matrix (GLSZM) Features

A Gray Level Size Zone (GLSZM) describes gray level zones in a ROI, which are defined as the number of connected voxels that share the same gray level intensity. A voxel is considered connected if the distance is 1 according to the infinity norm (26-connected region in a 3D, 8-connected region in 2D). In a gray level size zone matrix ***P(i,j)*** the **(i,j)^th^** element equals the number of zones with gray level ***i*** and size ***j*** appear in image. Contrary to GLCM and GLRLM, the GLSZM is rotation independent, with only one matrix calculated for all directions in the ROI.

**Let:**

**N_g_** be the number of discreet intensity values in the image

**N_s_** be the number of discreet zone sizes in the image

**N_p_** be the number of voxels in the image

**N_z_** be the number of zones in the ROI, which is equal to $\sum_{i=1}^{N_{g}} \sum_{j=1}^{N_{s}} \text{P}(i,j)$ and ***1 ≤ N_z_ ≤ N_p_***

***P(i,j)*** be the size zone matrix

***p(i,j)*** be the normalized size zone matrix, defined as $p(i,j)=\frac{\text{P}(i,j)}{N_{z}}$

***ϵ*** is an arbitrarily small positive number (≈2.2×10−16).

**Table S5.** **Detailed information about the GLSZM features in the final 9 radiomics features.**

| **Radiomic feature** | **Interpretation** | |
| --- | --- | --- |
| $\text{Large Area High Gray Level Emphasis}=\frac{\sum_{i=1}^{N_{g}} \sum_{j=1}^{N_{s}} \text{P}(i,j)i^{2}j^{2}}{N_{z}}$ | | Large Area High Gray Level Emphasis measures the proportion in the image of the joint distribution of larger size zones with higher gray-level values. |
| $\text{Zone Entropy}=-\sum_{i=1}^{N_{g}} \sum_{j=1}^{N_{s}} p(i,j)\log_{2}(p(i,j)+\epsilon)$ | | Zone Entropy measures the uncertainty/randomness in the distribution of zone sizes and gray levels. A higher value indicates more heterogeneneity in the texture patterns. |
| Adapted from http://pyradiomics.readthedocs.io/en/latest/features.html. | | |
