# Supplementary figures and images for: Radiomics for identifying lung adenocarcinomas with predominant lepidic growth manifesting as large pure ground-glass nodules on CT images
Source: PLoS One. 2022 Jun 24;17(6):e0269356. doi: 10.1371/journal.pone.0269356 (PMC9231804; doi:10.1371/journal.pone.0269356)

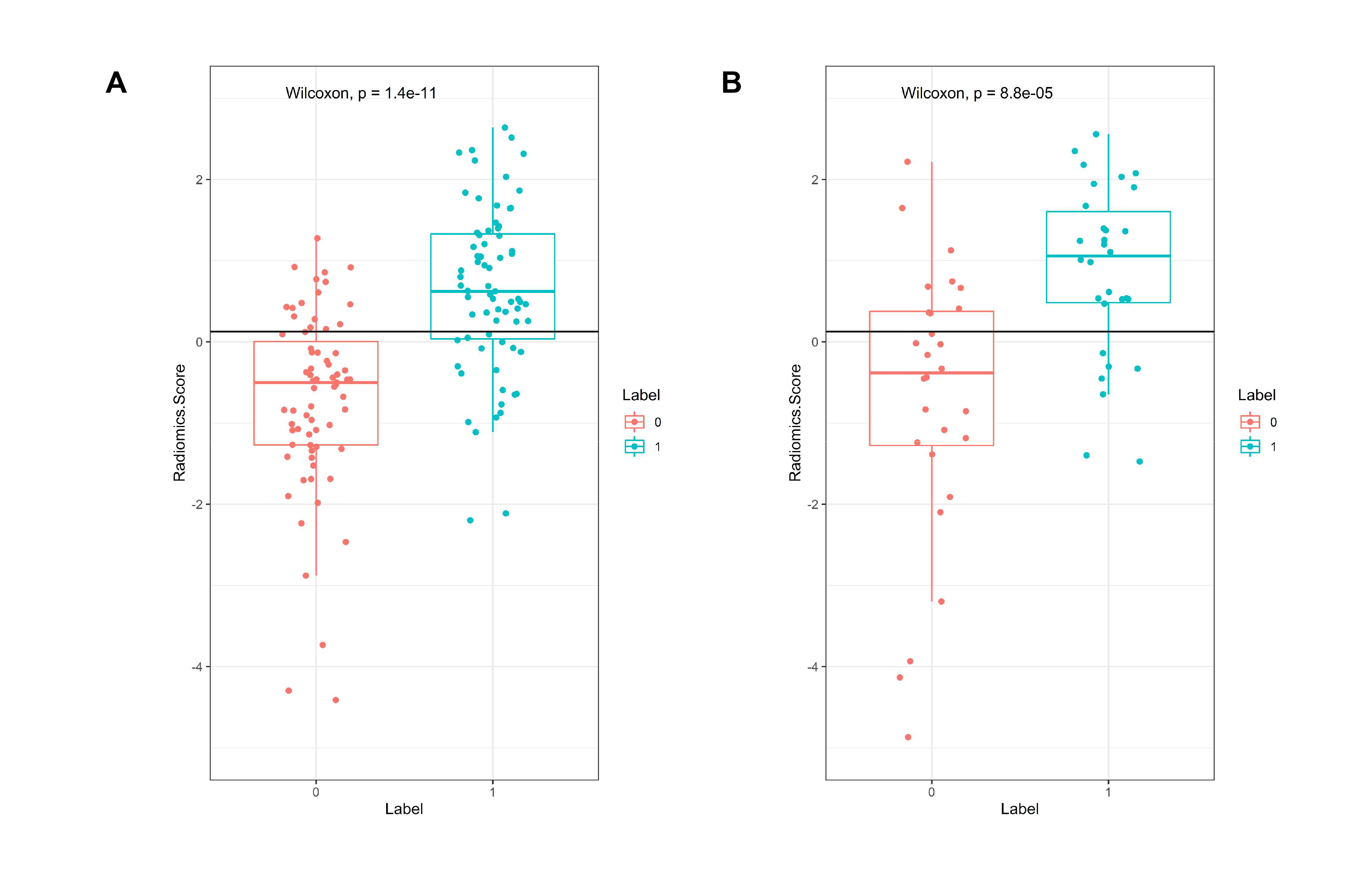

Supplement: S1 Fig — (TIF) [file pone.0269356.s003.tif]
