# Supplementary material for: Radiomics for identifying lung adenocarcinomas with predominant lepidic growth manifesting as large pure ground-glass nodules on CT images
Source: PLoS One. 2022 Jun 24;17(6):e0269356. doi: 10.1371/journal.pone.0269356 (PMC9231804; doi:10.1371/journal.pone.0269356)
Supplement: S1 Table — (DOCX) [file pone.0269356.s004.docx]

**S1 Table. Prediction performance of radiomics models based on six machine learning methods.**

| **Model** | **Training cohort** |  |  |  | **Test cohort** |  |  |  |
| --- | --- | --- | --- | --- | --- | --- | --- | --- |
|  | **AUC (95% CI)** | **SEN** | **SPE** | **ACC** | **AUC (95% CI)** | **SEN** | **SPE** | **ACC** |
| **LR** | 0.833 (0.768, 0.899) | 0.736 | 0.819 | 0.778 | 0.804 (0.692, 0.917) | 0.800 | 0.766 | 0.783 |
| **SVM** | 0.808 (0.744, 0.868) | 0.761 | 0.789 | 0.732 | 0.773 (0.669, 0.870) | 0.694 | 0.677 | 0.710 |
| **NBC** | 0.793 (0.734, 0.854) | 0.754 | 0.704 | 0.803 | 0.654 (0.535, 0.767) | 0.581 | 0.677 | 0.484 |
| **KNN** | 0.814 (0.756, 0.871) | 0.732 | 0.761 | 0.804 | 0.697 (0.579, 0.810) | 0.710 | 0.645 | 0.774 |
| **DT** | 0.866 (0.816, 0.913) | 0.838 | 0.901 | 0.775 | 0.660 (0.552, 0.770) | 0.661 | 0.645 | 0.677 |
| **RF** | 0.896 (0.850, 0.938) | 0.845 | 0.873 | 0.817 | 0.718 (0.609, 0.823) | 0.645 | 0.677 | 0.613 |

LR, logistic regression; SVM, support vector machine; NBC, naive Bayesian classifier; KNN, K-nearest neighbor; DT, decision tree; RF, random forest; AUC, area under the receiver operating characteristic curve; CI, confidence interval; SEN, sensitivity; SPE, specificity; ACC, accuracy.
