# Supplementary material for: Radiomics for identifying lung adenocarcinomas with predominant lepidic growth manifesting as large pure ground-glass nodules on CT images
Source: PLoS One. 2022 Jun 24;17(6):e0269356. doi: 10.1371/journal.pone.0269356 (PMC9231804; doi:10.1371/journal.pone.0269356)
Supplement: S2 Table — (DOCX) [file pone.0269356.s005.docx]

**S2 Table.** **Comparison of CT morphological and quantitative parameters between MIA/LPA and NLPA in the** **external validation cohort.**

|  | **MIA/LPA (N = 33)** | **NLPA (N = 16)** | ***p* value** |
| --- | --- | --- | --- |
| **Tumor location** |  |  | 0.394 |
| Left lower lobe | 5 (15.15%) | 6 (37.50%) |  |
| Left upper lobe | 12 (36.36%) | 3 (18.75%) |  |
| Right lower lobe | 1 (3.03%) | 1 (6.25%) |  |
| Right middle lobe | 6 (18.18%) | 2 (12.50%) |  |
| Right upper lobe | 9 (27.27%) | 4 (25.00%) |  |
| **Shape** |  |  | 0.520 |
| Irregular | 9 (27.27%) | 6 (37.50%) |  |
| Round and oval | 24 (72.73%) | 10 (62.50%) |  |
| **Tumor-lung interface (clear)** |  |  | 0.402 |
| Clear | 27 (81.82%) | 15 (93.75%) |  |
| Unclear | 6 (18.18%) | 1 (6.25%) |  |
| **Lobulation** |  |  | 0.300 |
| Presence | 24 (72.73%) | 14 (87.50%) |  |
| Absent | 9 (27.27%) | 2 (12.50%) |  |
| **Vacuole** |  |  | 0.130 |
| Presence | 4 (12.12%) | 5 (31.25%) |  |
| Absent | 29 (87.88%) | 11 (68.75%) |  |
| **Air bronchogram** |  |  | 0.180 |
| Presence | 10 (30.30%) | 8 (50.50%) |  |
| Absent | 23 (69.70%) | 8 (50.50%) |  |
| **Pleural indentation** |  |  | 0.898 |
| Presence | 13 (39.39%) | 6 (37.50%) |  |
| Absent | 20 (60.61%) | 10 (62.50%) |  |
| **Volume (cm^3^)** | 0.55 (0.35, 1.35) | 0.65 (0.54, 0.94) | 0.406 |
| **Maximum diameter (cm)** | 1.34 (1.10, 1.66) | 1.39 (1.18, 1.55) | 0.815 |
| **mCTv (HU)** | –687.43 ± 61.70 | –628. 34 ± 58.17 | 0.011 |
| **mCTv-Lcs (HU)** | –674.43 ± 67.31 | –619.86 ± 67.74 | 0.002 |
| **Mass (mg)** | 162.55 (120.45, 352.90) | 238.62 (203.04, 342.72) | 0.141 |

The values are presented as no. (%), mean ± standard deviation, or median (interquartile range). MIA, minimally invasive adenocarcinoma; LPA, lepidic predominant adenocarcinoma; NLPA, non-lepidic predominant adenocarcinoma; mCTv, mean CT value of the whole nodule; mCTv-Lcs, mean CT value of the largest cross-section; HU, Hounsfield units.
