# Supplementary material for: Radiomics for identifying lung adenocarcinomas with predominant lepidic growth manifesting as large pure ground-glass nodules on CT images
Source: PLoS One. 2022 Jun 24;17(6):e0269356. doi: 10.1371/journal.pone.0269356 (PMC9231804; doi:10.1371/journal.pone.0269356)
Supplement: S3 Table — (DOCX) [file pone.0269356.s006.docx]

**S3 Table. Comparison of frozen section and final pathology diagnosis.**

|  | **Frozen section diagnosis** | | |
| --- | --- | --- | --- |
| **Final pathology diagnosis** | **AIS** | **MIA** | **IA** |
| MIA (51) | 8 | 39 | 4 |
| LPA (51) | 0 | 4 | 47 |
| NLPA (102) | 2 | 9 | 91 |

The number of cases in each category is shown in brackets. AIS, adenocarcinoma in situ; MIA, minimally invasive adenocarcinoma; LPA, lepidic predominant adenocarcinoma; NLPA, non-lepidic predominant adenocarcinoma.
